# Pyrosequencing Reveals High-Temperature Cellulolytic Microbial Consortia in Great Boiling Spring after *In Situ* Lignocellulose Enrichment

**DOI:** 10.1371/journal.pone.0059927

**Published:** 2013-03-29

**Authors:** Joseph P. Peacock, Jessica K. Cole, Senthil K. Murugapiran, Jeremy A. Dodsworth, Jenny C. Fisher, Duane P. Moser, Brian P. Hedlund

**Affiliations:** 1 School of Life Sciences, University of Nevada, Las Vegas, Nevada, United States of America; 2 Division of Earth and Ecosystem Sciences, Desert Research Institute, Las Vegas, Nevada, United States of America; Université Paris Sud, France

## Abstract

To characterize high-temperature cellulolytic microbial communities, two lignocellulosic substrates, ammonia fiber-explosion-treated corn stover and aspen shavings, were incubated at average temperatures of 77 and 85°C in the sediment and water column of Great Boiling Spring, Nevada. Comparison of 109,941 quality-filtered 16S rRNA gene pyrosequences (pyrotags) from eight enrichments to 37,057 quality-filtered pyrotags from corresponding natural samples revealed distinct enriched communities dominated by phylotypes related to cellulolytic and hemicellulolytic *Thermotoga* and *Dictyoglomus*, cellulolytic and sugar-fermenting *Desulfurococcales*, and sugar-fermenting and hydrogenotrophic *Archaeoglobales*. Minor enriched populations included close relatives of hydrogenotrophic *Thermodesulfobacteria*, the candidate bacterial phylum OP9, and candidate archaeal groups C2 and DHVE3. Enrichment temperature was the major factor influencing community composition, with a negative correlation between temperature and richness, followed by lignocellulosic substrate composition. This study establishes the importance of these groups in the natural degradation of lignocellulose at high temperatures and suggests that a substantial portion of the diversity of thermophiles contributing to consortial cellulolysis may be contained within lineages that have representatives in pure culture.

## Introduction

Growing human populations and expanding industrialization have led to an increasing global demand upon finite supplies of fossil fuels, prompting growing interest in alternative fuel sources. Liquid biofuels that are compatible with modern vehicles and the extant fuel delivery and supply infrastructure, such as bioethanol, are an appealing supplement to petroleum-based fuel supplies [1]. However, current bioethanol production methods, considered “first-generation” biofuel technology, rely on fermentable sugars from plants traditionally utilized as food crops, so their production directly competes with the supply of food for human populations [2]–[4] and contribute to a range of coincident environmental concerns such as soil erosion, loss of biodiversity, and impact on water resources [5]–[7]. The negative consequences of existing biofuel technologies have stimulated interest in the development of so-called “second-generation” biofuel technologies, which derive fermentable sugars from dedicated crops or lignocellulosic waste produced by agriculture, forestry, and other industries.

The structural complexity and low aqueous solubility of lignocellulosic biomass creates a significant barrier to its use and ethanol production from lignocellulosic sources is currently cost-prohibitive [8]. Existing production methods involve chemical, thermal, and mechanical pretreatment of plant tissues to increase the availability of the structural carbohydrates for hydrolysis, followed by saccharification by cellulolytic microorganisms or their purified cellulases. However, the high unit cost of cellulases has limited their application to ethanol production, stimulating a demand for more cost-effective enzymes that would make second-generation biofuels an economically feasible alternative to fossil fuels and first-generation biofuels [9]. Thermostable cellulases offer several potential benefits to mitigate the high costs of enzymatic saccharification of lignocellulose. They tend to have much greater activity at their optimal temperature than those from mesophilic organisms because each 10°C increase in reaction temperature increases enzymatic rates two- to three-fold [10]. Higher reaction temperatures also increase the solubility of substrates, increasing the yield of the end products [10], and reduce the viscosity of the reaction mixture, decreasing water demand [11]. Additionally, thermostable enzymes are resistant to denaturation from other factors and highly stable for long-term storage, lengthening their shelf life and operational life during lignocellulose digestion [10], [12].

A number of studies have focused on the isolation and characterization of cellulolytic thermophiles as possible sources of cellulases for the biofuels industry (reviewed in [13]). In terrestrial geothermal systems, the majority of known cellulolytic thermophiles belong to the bacterial phyla *Firmicutes*, *Thermotogae*, and *Dictyoglomi* and the archaeal order *Desulfurococcales*. Several *Firmicutes* use crystalline cellulose and other polymers for biomass substrates. For example, a variety of *Caldicellulosiruptor* species with temperature optima of 70 to 78°C can utilize cellulose with varying degrees of crystallinity [13]–[15]. The archaeon *Desulfurococcus fermentans,* which grows optimally between 80 and 82°C, currently delineates the known high temperature limit for crystalline cellulose degradation by a pure culture [16]. In contrast to the *Firmicutes* and *Desulfurococcales*, the *Thermotogales* are not known to degrade crystalline cellulose. However a variety of *Thermotogales* species with optimal growth temperatures ranging from 65 to 80°C use hemicellulose, α- and β-linked glucans, and pectin as carbon and energy sources [13], [17], [18]. Finally, the two species of *Dictyoglomus*, *D. turgidum* and *D. thermophilum,* can depolymerize xylan and other polymers with optimal temperatures in the range of 72 to 78°C [19], [20].

Despite the solid foundation provided by studies of pure cultures of cellulolytic thermophiles, a well-known challenge in environmental microbiology is the elucidation of the metabolic capabilities and ecological roles of the majority of microorganisms that defy laboratory cultivation (reviewed in [21]). To gain insight into the structure of natural cellulolytic communities and access to yet-uncultivated microorganisms, a number of recent studies have taken a cultivation-independent approach to study high temperature cellulolysis by sequencing 16S rRNA genes or metagenomes from thermophilic communities acting upon lignocellulosic materials. Two studies focusing on terrestrial compost systems degrading lignocellulosic substrates at 60°C revealed enriched communities dominated by *Paenibacilli*, *Rhodothermus*, and *Thermus,* and showed that changes in the feedstock led to community-level responses [22], [23]. Another study of a switchgrass-degrading bioreactor with temperature cycled up to 54°C documented enrichment of a variety of *Firmicutes* and a few phylotypes in the *Chloroflexi*, *Proteobacteria*, and *Actinobacteria* [24]. Despite the potential for high-temperature communities to serve as sources of novel cellulases, no such studies have explored the composition and structure of cellulolytic microbial communities at higher temperatures. It is well-known that microbial communities in >75°C habitats are distinct from those at lower temperatures, even at the phylum level [25]–[28], and therefore the potential for applied and basic scientific discovery resulting from the investigation of cellulolytic communities in high-temperature environments such as terrestrial hot springs is significant.

As a first step towards bridging this fundamental knowledge gap, we established a series of cellulosic enrichments in Great Boiling Spring (GBS), Nevada. GBS is a large, circumneutral hot spring located in the U.S. Great Basin. The rate of sinter precipitation around the perimeter of GBS is low [29], allowing for plant growth up to the edge of the spring, which provides a regular influx of allochthonous lignocellulosic material into the spring. This spring has also been found to harbor a rich assortment of novel microorganisms, with significant portions of the sediment microbial community members of the candidate phyla “*Aigarchaeota*”, GAL35, and GAL15 [26], [29]–[32]. We established eight *in situ* enrichments in GBS, each containing one of two different lignocellulosic substrates, in both the sediment and water column of the source pool and outflow channel of the spring. 16S rRNA gene pyrosequencing methodologies were then employed to characterize the microbial communities that colonized the enrichments and to compare them to those in corresponding sediment samples to examine the effect of lignocellulosic enrichment in a natural high-temperature setting and better understand lignocellulose-degrading organisms and communities.

## Materials and Methods

### Sample Site, Permits, Incubation, and Collection

**Figure 1:**
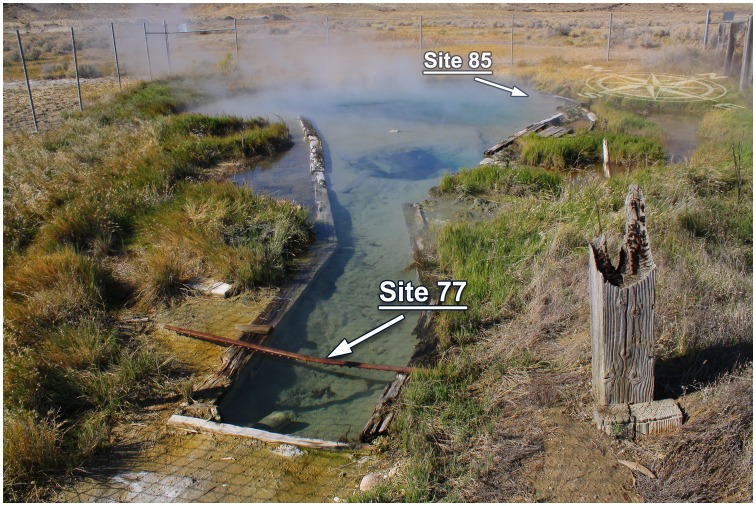
Photograph of GBS with sample incubation sites, Site 85 and Site 77, indicated.

GBS is located on private land at N40° 39.684′ W119° 21.978′ near the town of Gerlach, Nevada, at the edge of Pleistocene Lake Lahontan (Figure 1). The site is the focus of long-term research projects with support and permission from the land owner, and no formal permit is required. “Site 85”, denoted for its average water temperature ∼85°C, is at the northwest side of the main spring pool (N40° 39.686′ W119° 21.979′). “Site 77”, average water temperature ∼77°C, is in the outflow and ∼11 m from Site 85 (N40° 39.682′, W119° 21.973′). Site 77 averaged 8.3°C (range: 6.2–14.1°C; standard deviation: 1.4°C) cooler than Site 85 during the incubation period. Site 85 corresponds to Site A and Site 77 to Site C, as described by Hedlund *et al.* [33] and Cole *et al.* [26].

Enrichment packets were prepared by enclosing 20 g of either aspen shavings, commercially available as pet litter (Kaytee Products, Chilton, WI), or ammonia fiber explosion (AFEX)-treated corn stover (kindly provided by Bruce Dale, Michigan State University). Each packet was constructed by sewing together two ∼10 cm squares of 100-µm pore size nylon mesh (#NM0100P3 Pentair Industrial, Hanover Park, IL) together with nylon thread. Sediment incubations were buried approximately 1 cm below the sediment-water interface. Water column incubations were suspended ∼10 cm below the air-water interface, enclosed within 20×12×5 cm polypropylene boxes punctured with ∼100 0.5 cm holes to maintain position and allow water exchange. All packets were deployed on 29 August, 2009. Those at Site 77 were harvested 1 November, 2009 and those at Site 85 were harvested 29 November, 2009. The incubations were terminated on different dates because changes consistent with lignocellulolysis were not visibly evident in the feedstocks incubated at Site 85 on the planned harvest date. Immediately after retrieval, the contents of each packet were aseptically divided into sterile, conical, polypropylene tubes using sterilized forceps. The subsamples were then frozen on dry ice for transport and stored at −80°C prior to analysis. Non-incubated aspen shavings and corn stover were prepared for DNA extraction and quantification by soaking ∼5 g of each substrate in 50 ml conical tubes containing 40 ml 0.5× TE buffer (5 mM Tris, 0.5 mM EDTA, pH 8) at 80°C for 4 hours. The buffer was decanted and the remaining wetted substrate stored at −80°C.

### Natural Water and Sediment Sample Collection

Collection of the natural water and sediment samples was described in detail by Cole *et al* [26]. Briefly, sterile, 50 ml conical polypropylene tubes were used to collect the top ∼1 cm of sediment at each site for natural sediment samples. Sediment samples were collected at Site 85 on three dates between June 2009 and July 2010 and Site 77 on two dates in February 2010 and July 2010. Additionally, bulk water samples were collected from the main spring pool near Site 85 by tangential flow filtration (Prep/Scale filter with 30 kDa molecular weight cut-off, Millipore, Billerica, MA, USA) or 0.2 µm normal filtration (Supor filter, hydrophilic polyethersulfone, Pall Corporation, Port Washington, NY) on three dates between June 2006 and February 2010.

### Environmental Data Collection

The water temperature and pH at each site was measured at the beginning and end of incubation with a handheld pH 5 meter (LaMotte, Chestertown, MD). Temperature data logger iButtons (DS1922T; Maxim, Sunnyvale, CA), set to record temperature every 2 hours, were sealed in 50 ml conical tubes and suspended in the spring water at the two incubation sites from September 15, 2009 until the end of incubation (Table 1, Figure S1).

**Table 1:** Sample incubation conditions and pyrotag yields.

Sample Name	Avg. Temp. (°C)^a^	Site	Enrichment	Incubation Location	N_i_ c	N_f_ d
UW	81	Water	None	na^b^	na^b^	11,233
U77	74	77	None	na^b^	na^b^	11,308
U85	83	85	None	na^b^	na^b^	14,516
77AS	77	77	Aspen	Sediment	29,188	21,006
77AW	77	77	Aspen	Water	25,528	18,924
77CS	77	77	Corn Stover	Sediment	17,266	12,128
77CW	77	77	Corn Stover	Water	14,527	10,565
85AS	85	85	Aspen	Sediment	7,094	4,842
85AW	85	85	Aspen	Water	12,167	8,259
85CS	85	85	Corn Stover	Sediment	20,416	12,868
85CW	85	85	Corn Stover	Water	32,241	21,349

a Average temperature of natural samples or as recorded during incubation (Figure 1).

b na, not applicable.

c Number of pyrotags generated by pyrosequencing.

d Number of quality-filtered pyrotags used in analysis.

### Substrate Lignocellulose Content Analysis

All substrate content analyses were carried out by Dairy One, Inc. of Ithaca, New York. Acid detergent fiber (ADF) content was determined by digesting 0.5 g samples in Ankom Technology FAD20CB acid detergent solution (2.93% w/v sulfuric acid and 2% w/v cetyltrimethylammonium bromide; Ankom Technology, Macedon, NY) for 75 minutes in an Ankom A200 digestion unit. Samples were rinsed three times in boiling water for five minutes, soaked in acetone for three minutes, and then dried at 100°C for two hours. Samples were weighed before and after digestion to determine ADF content.

Lignin and cellulose content were determined by digesting the ADF residue for three hours in an Ankom Daisy II incubator at ambient temperature, using 72% w/w sulfuric acid. After rinsing and drying, the remaining solid was considered the lignin portion of the original sample. The portion dissolved away by this treatment was considered cellulose.

Neutral detergent fiber (NDF) content was determined by digesting 0.5 g samples for 75 minutes in an Ankom A200 digestion unit with Ankom Technology FND20 neutral detergent solution (3.0% sodium lauryl sulfate, 1.86% EDTA disodium dehydrate, 0.68% sodium borate decahydrate, 0.46% anhydrous dibasic sodium phosphate, and 1.0% triethylene glycol), amended with 0.2% α-amylase and 1% w/v sodium sulfite. Samples were then rinsed twice for five minutes with a boiling α-amylase solution, once for five minutes with boiling water, soaked in acetone for 3 minutes, and dried at 100°C for two hours. Samples were weighed before and after digestion to determine NDF content.

Hemicellulose content was calculated by subtracting the ADF content from the NDF portion.

Ash content was determined according to the Association of Official Analytical Chemists (AOAC) Official Method 942.05. Pre-weighed samples were held at 600°C for two hours to incinerate organic materials. The remaining mass after treatment was weighed to determine the ash portion of the original sample.

### DNA Extraction, PCR Amplification, and Pyrosequencing

DNA extractions were performed on ∼0.5 g portions of incubated material using the FastDNA Spin Kit for Soil (MP Biomedicals, Solon, OH) as described previously [34]. DNA was extracted from each portion, pooled per sample, and precipitated with 70% ethanol. DNA was resuspended in sterile 0.5×TE (5 mM Tris, 0.5 mM EDTA, pH 8) and quantified using a Nanodrop 1000 (Thermo Scientific, Waltham, MA, USA).

Extracted DNA from all samples was shipped on dry ice to the United States Department of Energy’s Joint Genome Institute (JGI, Walnut Grove, CA) for pyrosequencing. The V8 and a portion of the V7 hypervariable regions of the 16S rRNA gene were amplified using primers 926F454TitFNew (AAA CTY AAA KGA ATT GRC GG) and 1392R (ACG GGC GGT GTG TRC) and sequenced using 454 GS-FLX Titanium pyrosequencing (454 Life Sciences, Branford, CT, USA), as described by Cole *et al.* [26].

### Pyrotag Data Filtering and Preparation

146,162 16S rRNA gene pyrosequence fragments (pyrotags) from eight natural samples and 158,427 pyrotags from eight enrichment samples were trimmed short of their first ambiguous base. Sequences shorter than 120 nt, including 5 nt multiplex ID barcodes and primers, were excluded from further processing. Quantitative Insights into Microbial Ecology (QIIME) software package v 1.5.0 [35] was used to denoise sequences and all pyrotags were truncated to a maximum length of 204 nt, based on minimum quality scores. OTUs at 97%, 95%, 92%, 85%, and 80% minimum identity levels were identified by QIIME, using the uclust algorithm, and the most abundant representative sequence within each OTU was inspected for chimeras with ChimeraSlayer [36] against the NAST-aligned “gold” alignment file supplied with the ChimeraSlayer package using QIIME. Each OTU was BLASTed against the Greengenes database [37] and assigned the most detailed taxonomy that matched ≥90% of the sequences within a given OTU. Sequences that could not be assigned taxonomic identification were investigated using the NCBI BLAST database using the megablast algorithm [38]. Eukaryotic sequences and potential chimeras were omitted from further processing. The GAL35 lineage was removed from the OP1 phylum and maintained as its own phylum, consistent with previous versions of the Greengenes phylogeny database. Sequences, taxonomies, and OTU definitions were imported into a MySQL Community Edition version 5.5.24 database [39].

Instead of comparing the enriched communities to only a single natural sample from each site, a generalized community was created *in silico* to serve as the representative natural community for each sampling site. The use of these aggregate samples minimized the effects of short-term community fluctuations in the sediment microbial community due to temperature variations in GBS [26]. Three sets of sequences (UW, U85, and U77) representative of the natural sediment and water communities at each sampling site were created by randomly subsampling and combining equal proportions of the curated pyrotag datasests obtained from natural samples collected at each site and described by Cole *et al.* [26]. Sample UW consisted of one third of each of the natural water samples collected, Sample U85 consisted of one third of each of the sediment samples collected at Site 85, and Sample U77 consisted of one half of each of the sediment samples collected at Site 77. These aggregate samples were exported from the database as QIIME-compatible OTU tables for community analyses. It is worthwhile to note that the aggregation of natural samples was necessary for the statistical framework employed here and that none of the individual natural samples resembled any of the enriched communities [26].

### Community Composition Statistical Analyses

QIIME was used to calculate OTUs observed (S) and Simpson’s Index of Diversity (1-D) [40] at each OTU identity level measured. Simpson’s index of evenness was calculated in Microsoft Excel 2010, based on S and 1-D.

A subsample of 4,842 pyrotags from each sample was identified by random sorting in MySQL to produce a pyrotag subset, with all samples rarefied to match the smallest sample (85AS). The rarefied data were exported and QIIME was used to calculate the Bray-Curtis dissimilarity scores [41] between each rarefied sample. The Bray-Curtis dissimilarities were used within QIIME to produce a sample cluster tree and principal coordinates (PCoA) graph. Permutational multivariate analysis of variance (PERMANOVA; also called non-parametric MANOVA, NPMANOVA) was used to calculate the statistical significance of community composition differences between groups of samples [42]. PERMANOVA statistics were calculated for sample groups identified by the cluster tree and for groups separated by experimental conditions using PAST v. 2.11 [43], based on Bray-Curtis dissimilarity with 9,999 permutations. For groups that were found to be significantly different, PAST was used to perform SIMPER analysis to identify the most discriminatory OTUs between the sample groups.

### Enrichment Calculation

Genus fold enrichment was calculated in MySQL. All samples were normalized by multiplying the total number of sequences observed (hits) per genus in each sample by a sample-specific multiplier in order to increase each sample to match the largest sample (85CW: 21,349 pyrotags). One hit was then added to each genus in every sample in order to eliminate divide-by-zero errors. Adjusted genus hits in enrichment samples were then divided by the adjusted genus hits in the corresponding natural sediment sample in order to calculate approximate fold enrichment. This technique slightly underestimated actual enrichment, especially for low-abundance genera. The base-10 logarithm of the fold enrichment ratios for highly-abundant genera and specific OTUs of interest were exported from the database. The exported file was imported into the R statistical package [44], which was used to produce a heatmap of enrichment.

SFF files containing the original unfiltered pyrosequences were submitted to the NCBI Sequence Read Archive (77AS, SRX203068; 77AW, SRX203070; 77CS, SRX203071; 77CW, SRX203074; 85AS, SRX203075; 85AW, SRX203076; 85CS, SRX203078; 85CW, SRX203079) and associated with NCBI BioSample SUB120753and NCBI BioProject SUB112230.

## Results and Discussion

### Lignocellulose Enrichments in Great Boiling Spring

Because of its large size, topography, and low flow rate, GBS contains temperature zones that differ by more than 20°C [30]. For this study, two sites (Site 85 and Site 77) along the perimeter of GBS were selected for lignocellulosic substrate incubation and sample collection (Table 1, Figure 1). Long-term temperature loggers were deployed during incubation to record the temperature at each incubation site (Figure S1). “Site 85” was close to the geothermal source and had an average temperature of ∼85°C, approximately equal to the source water. “Site 77” was near the spring’s outflow and had an average temperature of ∼77°C.

Two packets of aspen shavings (samples designated with “A”) and AFEX-treated corn stover (“C”) were incubated at each site. One packet of each substrate was buried in the sediment (“S”) and the other was suspended in the water column (“W”). Each of the eight enrichment samples is indicated here by the incubation site, substrate type, and incubation location, *e.g.* 85CS is corn stover incubated in the spring sediment at Site 85 (85CS = Site 85, Corn stover, Sediment). The microbial communities enriched on the lignocellulose were compared with aggregate samples (samples U85, U77, and UW) created *in silico* to represent the natural communities of GBS at each site. Aggregate samples U77 and U85 represented the natural sediment communities at Site 77 and Site 85, respectively, and UW represented the natural community of the bulk water of GBS. The enrichment packets suspended in the aerobic spring water developed primarily anaerobic communities very similar to those in the sediment enrichments (Figure S2), likely due to microbial respiration exceeding the diffusion of oxygenated water into the compact material in the packets. Therefore, all enrichment samples were compared to the natural sediment sample at the respective site.

### Evidence of Microbial Growth and Effects of Incubation on Composition of Lignocellulosic Materials

To confirm growth on the lignocellulosic substrates, we quantified the DNA in non-incubated cellulosic material and in incubated samples. Post-incubation DNA yields were higher compared to non-incubated samples for both corn stover (2.2 to 5.7-fold increase) and aspen (12.7 to 22.3-fold increase) substrates (Table S1), indicating an increase in biomass and enrichment on the cellulosic materials *in situ*.

**Figure 2:**
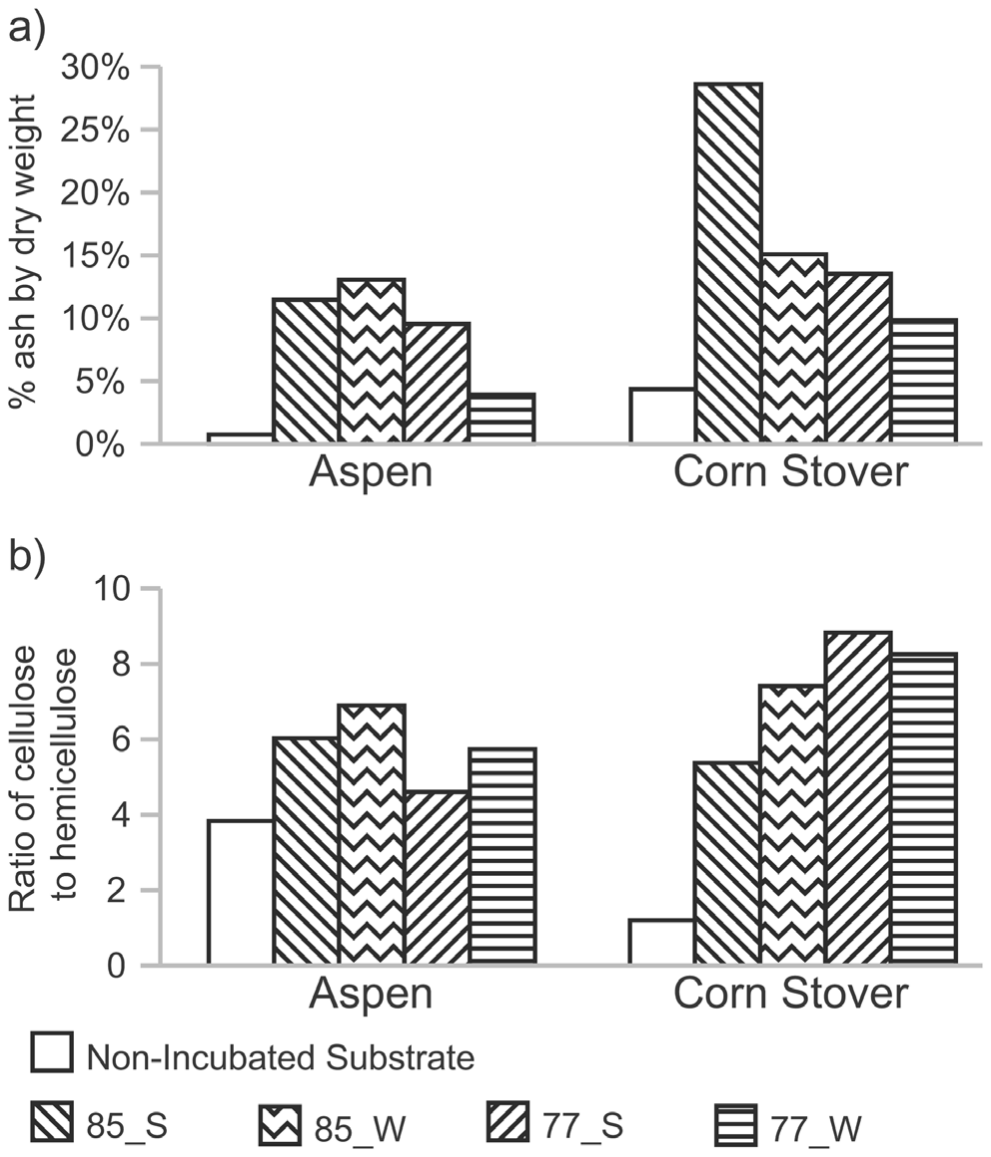
Composition of non-incubated and incubated lignocellulosic substrates. (a) Ash content. (b) Cellulose to hemicellulose ratio.

The material used in the enrichments was composed mainly of fiber (cellulose, hemicellulose, and lignin), with smaller amounts of non-combustible material (ash), protein, and bioavailable sugars (Table S2). Differences in the composition of incubated and unincubated control substrates were used to infer microbial community activity; however, some caution is warranted since changes due to biological activity cannot be distinguished from those due to aqueous solubilization or other abiotic processes. The change in ash content (mineral content) was used as a proxy for organic matter consumption, with a greater proportion of ash remaining after incubation corresponding to greater consumption of organic material. The ash content was higher in all samples post-incubation (1.3 to 16.4-fold increase) than in non-incubated controls, suggesting organic matter consumption (Figure 2a). Incubation also led to an increase in the ratio of cellulose to hemicelluloses (Figure 2b), indicative of preferential degradation of hemicellulose, which is consistent with the greater diversity and higher growth temperature optima of thermophiles able to digest hemicellulose as compared to cellulose [12]. This was particularly evident in the corn stover samples, where the cellulose to hemicellulose ratio increased from 1.2 in the non-incubated substrate to 5.4 to 8.8 in the samples incubated in the spring.

Lignin content was mostly unaffected in the aspen shaving enrichments, but decreased in all corn stover enrichments (Table S2). The cause of this difference is unclear. However, as lignin is covalently bonded to hemicellulose, the loss of lignin could be due to the degradation of hemicellulose as opposed to direct biological metabolism of lignin. No correlations between utilization of a particular component and either temperature or incubation location were observed.

### Microbial Community Diversity Changes in Response to Lignocellulose Enrichment

In order to assess the differences between the natural sediment communities and the cellulolytic communities, we analyzed operational taxonomic units (OTUs). OTUs were defined based on ≥97% pyrotag identity, which approximated the species level, although use of the V8 region of the 16S rRNA gene likely resulted in underestimations of the true richness [45]. Six hundred one OTUs were identified among all enrichment and natural sediment samples, 228 of which were found in the natural sediment communities and 460 of which were found in the enrichment samples. Only 87 OTUs were shared between enrichment samples and natural sediment communities and only 3 OTUs (#C236, #C600, and #C603) were found in greater than 5% relative abundance in the natural sediment community and any enrichment sample (Table S3), demonstrating the distinctness of enriched communities as compared with the natural communities.

All samples from Site 85 had a lower species richness than Site 77 samples, which is consistent with earlier findings of a temperature-driven richness gradient in GBS sediments [26]. Observed richness was similar in all samples at Site 85, including the aggregate natural sediment sample, 85 U. Three of the Site 77 enrichment samples had fewer OTUs than the natural sediment sample at that site, whereas the fourth had approximately the same richness as 77 U (Figure S3a). Similar results were observed from genus to phylum level and no significant pattern in richness was observed when comparing lignocellulosic substrate or sample incubation location (Figure S4a). No significant correlation was found between sequencing depth and species richness (R^2^ = 0.0999; p = 0.344), indicating that the observed differences in species richness represent actual differences in the samples, rather than artifacts of incomplete sampling.

Simpson’s index of evenness [40] was not significantly different in the natural sediment as compared with communities that developed due to lignocellulose enrichment (Site 85, p = 0.499; Site 77, p = 0.260; Figure S3b). Enrichments from Site 77 tended to be less even at all taxonomic levels than those from Site 85, although these differences were not significant (p = 0.188; Figure S4b).

### Comparison and Clustering of Sample Communities

**Figure 3:**
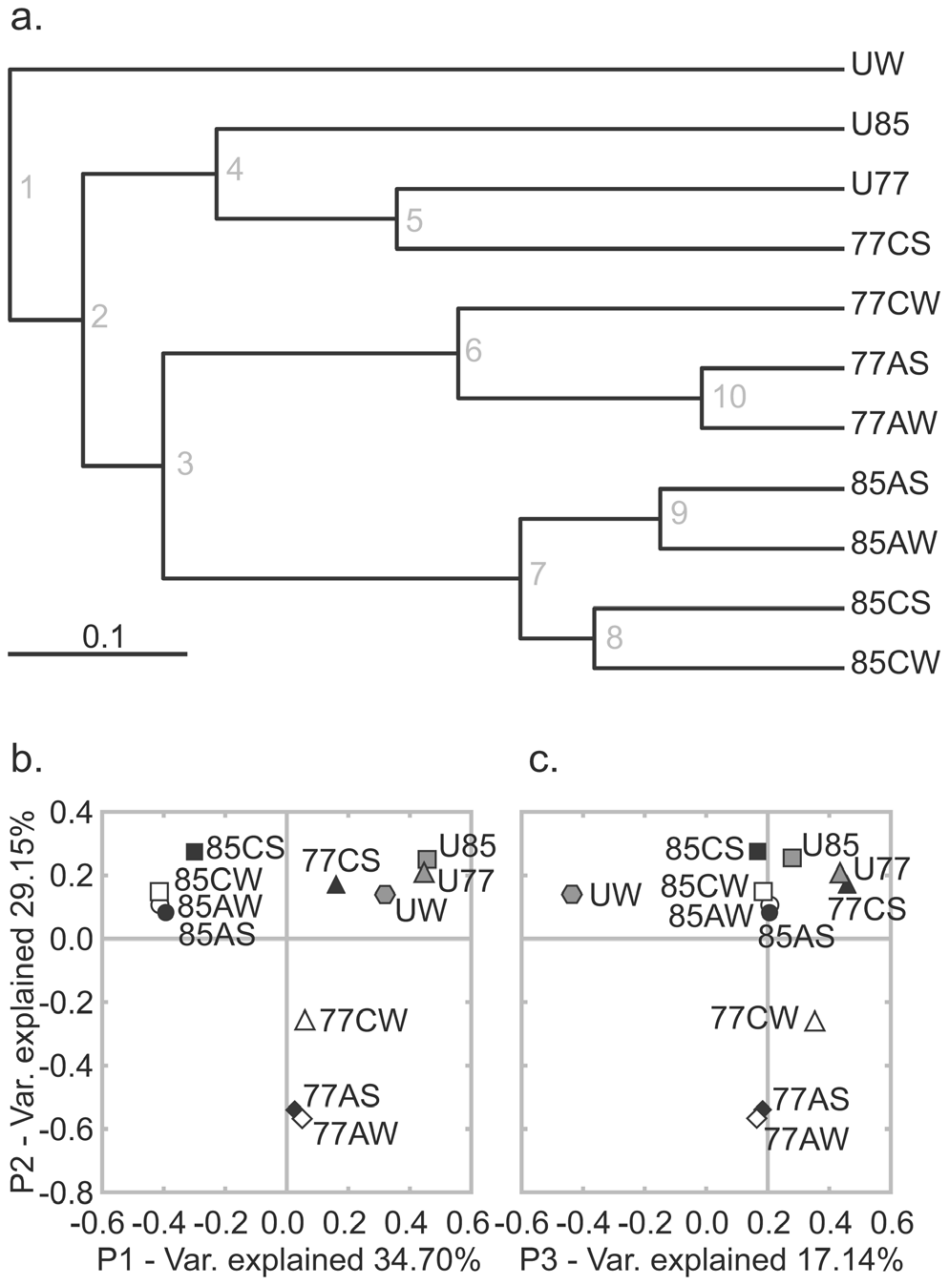
Natural and enriched samples clustered based on Bray-Curtis dissimilarity calculations of rarefied samples. (a) Cluster tree with samples grouped according to the similarity of the community composition of the samples. All nodes were supported by jackknife scores ≥99.9% after 1000 permutations. (b) PCoA of sample distances on principal coordinate 1 (P1) and principal coordinate 2 (P2), with a total of 63.85% of variation explained. (c) PCoA showing sample distances on principal coordinate 2 (P2) and principal coordinate 3 (P3), with a total of 46.29% of variation explained.

Hierarchical clustering and principal coordinate analysis (PCoA) of the enrichment and natural sediment samples revealed the enrichment samples to have community compositions distinct from the natural sediment communities, with the exception of 77CS (Figure 3). The most significant variable influencing community composition was whether the sample was natural or enriched with lignocellulose, as evidenced by node 2 in the cluster tree (Figure 3a) and the clustering of natural samples in the upper-right quadrant of the PCoA with P1 vs P2 (Figure 3b). Further segregation of samples was influenced by average incubation temperature, shown by node 3 (Figure 3a) and P2 of the PCoA (Figure 3b). Lastly, the type of lignocellulosic material in the sample influenced community composition, visible within nodes 6 and 7in the cluster tree (Figure 3b) and by clustering PCoAs (Figure 3b,c). Incubation of samples in the water column or sediment did not contribute significantly to any differences in community composition.

In order to test the significance of the variables influencing community composition, we performed PERMANOVA tests on all nodes in the sample cluster tree in Figure 3a that joined three or more samples and on groups defined by experimental conditions (Table S4). Tree node 2, which separated the enrichment samples from the natural sediment samples and 77CS, represented a highly significant division (p = 0.0084). The significance of lignocellulose enrichment was confirmed by a comparison of all enrichment samples (including 77CS) to the natural sediment samples (p = 0.0199). Incubation site/temperature was also confirmed as a significant factor in community composition, whether considering all enrichment and natural sediment samples at each site (p = 0.0152) or only enrichment samples (tree node 3; p = 0.0275). Neither the type of lignocellulosic material included in the enrichment packets, nor whether the packets were suspended in the bulk water or buried in the sediment, were significant (p = 0.1765, 0.8306, respectively).

77CS was anomalous in that it clustered with the natural Site 77 sediment sample, U77, rather than the other enrichment samples. 77CS was the least dissimilar sample from U77, with a Bray-Curtis dissimilarity score of 0.508. Despite its similarity to the natural samples based upon overall community composition, 77CS did show strong evidence of enrichment. Notably, *Thermotoga*, which was highly enriched in all samples, was the most abundant genus in 77CS and the nutritional analysis of 77CS suggested strong cellulolytic activity.

### Specific Microbial Taxa Enriched on Lignocellulosic Substrates

Similarity percentage (SIMPER) analysis was used to identify the organisms most responsible for the differences between the communities identified by PERMANOVA as significantly different (Tables S5, S6, S7, S8). Examination of OTUs identified by SIMPER to be the most discriminating between enrichments and natural sediment communities (Table 2) revealed that lignocellulose amendment led to significant enrichment of close relatives of the known cellulolytic and hemicellulolytic organisms *Thermotoga* (#C529, #C782) and *Dictyoglomus* (#C692), a potentially heterotrophic and hydrogenotrophic member of the *Archaeoglobaceae* (#C903), and an unidentified *Ignisphaera*-like archaeon (#C359). Conversely, there was a significant decrease in the relative populations of OTUs that dominated the natural sediment communities, such as candidate groups GAL35 (OTU #C603), “*Aigarchaeota*” (#C056, #C487; [46]), and NAG1 (#C136 [47]) and the genus *Aeropyrum* (#C199). It is important to note that, although many enriched microorganisms have a plausible role in consortial cellulolysis, it is likely that others do not. For example, some microorganisms might metabolize exopolysaccharides exuded by the primary cellulolytic organisms or simply colonize solid substrate without metabolizing it, as has been noted for *Thermotoga* colonizing inert substrates added to planktonic cultures in the laboratory [48].

**Table 2:** Significant OTUs discriminating between enrichment and natural samples.

OTU	Identity	Δ^a^	Contrib. (%)^b^	Natural Samples (%)^c^	Enrichment Samples (%)^d^
C529	*Thermotoga* sp.	+	10.76	0.04	19.25
C603	GAL35	−	10.36	21.89	3.47
C056	“*Aigarchaeota*”	−	9.18	16.67	0.28
C199	*Aeropyrum* sp.	−	6.22	11.61	0.51
C359	*Ignisphaera*-like	+	6.08	0.00	10.84
C782	*Thermotoga* sp.	+	5.81	0.10	10.41
C136	NAG1	−	3.97	7.10	0.06
C903	*Archaeoglobus* sp.	+	3.95	0.65	7.70
C692	*Dictyoglomus* sp.	+	3.66	0.13	6.61
C487	“*Aigarchaeota*”	−	3.62	6.46	0.42

a Difference between natural sediment and enrichment populations. +, OTU has greater representation in enrichment samples than natural samples. −, OTU has lower representation in enrichment samples than natural samples.

b Percent contribution to community composition difference.

c Average percent representation in natural sediment communities.

d Average percent representation in enrichment sample communities.

**Figure 4:**
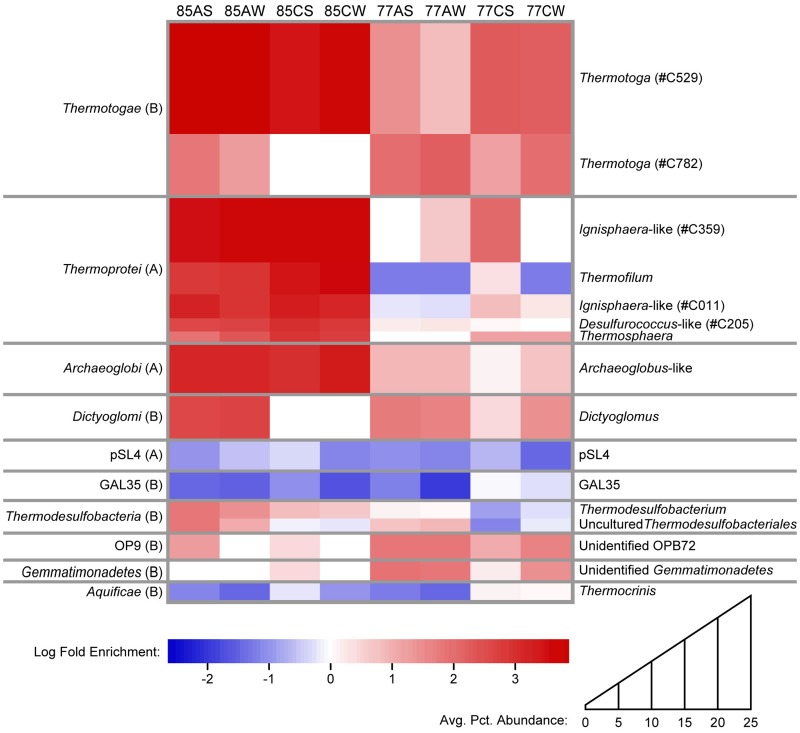
Heatmap showing log fold enrichment of highly abundant genera and OTUs of specific interest. Taxa are scaled vertically based on percent representation in all enrichment samples, as shown in average percent abundance key. Red, increased relative abundance over natural sediment community at same sampling site; white, no change; blue, decreased relative abundance.

Calculation of the fold enrichment of the genera identified within the samples (Figure 4) reinforced the distinctiveness of the communities formed within the enrichment packets. The most abundant genera in the enrichment samples (*Thermotoga*, *Ignisphaera*-like *Desulfurococcaceae*, *Archaeoglobus*-like *Archaeoglobaceae*, *Dictyoglomus*, and *Thermofilum*) were highly enriched over the natural samples. The genera that dominated the natural sediment communities (*Aeropyrum* and members of the uncultivated lineages “*Aigarchaeota*”, GAL35, and NAG1) showed significantly lower relative abundance in the enrichment sample populations.

The natural sediment communities of GBS consisted primarily of microbial lineages that have not been cultivated in the laboratory. Only 28.3% of the community in 85 U and 32.0% of the community in 77 U were comprised of organisms belonging to described families. The enrichment communities, however, averaged 77.2% identified at the family level (minimum 77CS, 58.5%; maximum 85CW, 97.2%; standard deviation 12.8%). The predominance of families with cultivated representatives in the lignocellulose-enriched communities may be due to the relative ease with which organisms with heterotrophic metabolisms are cultivated in the laboratory or the historical focus on cellulolytic organisms for biofuels-related studies.

### Enrichment of Highly Carbohydrate-active Thermotoga

The difference in the dominant OTUs between natural and lignocellulose-enriched communities was also reflected at broader taxonomic levels. Considering the relative abundance of the bacterial phyla and archaeal classes that comprised each community (Figure S2), *Thermotogae* represented at least 25% of each enrichment sample from Site 77 and three of the four enrichment samples from Site 85, but less than 1% of either natural sediment sample. Highly enriched *Thermotogae* consisted of two OTUs, #C529 and #C782. OTU #C529 was 100% identical to previously cultivated strains of *Thermotoga petrophila* and *T. naphthophila* and 99% identical to *T. maritima* and *T. neapolitana* over the pyrotag length. This OTU was highly enriched in all enrichment samples except 77AS and 77AW, showing 179- to 7480-fold enrichment over natural sediment communities and a relative abundance that ranged from 14.1% to 35.1% of the total enriched communities. The Site 77 aspen enrichment communities, where OTU #C529 was not abundant, had a high representation of OTU #C782, which shared 99% sequence identity with *T. thermarum* and 98% sequence identity with *T. hypogea*. OTU #C782 represented 22.0% to 33.6% of the Site 77 aspen enrichment communities and displayed a 101- to 154-fold increase over U77. OTU #C782 also represented 22.7% of the 77CW sample, but less than 4% of any other community.

No *Thermotoga* species has been shown to grow on crystalline cellulose, but some are known to grow on amorphous cellulose [17], [49] and a variety of other carbohydrate polymers, including other β-linked glucans, α-linked glucans, hemicellulose, and pectin [13]. The four cultivated *Thermotoga* species most similar to OTU #C529 share ≥99% identity over their full-length 16S rRNA sequences and 77 to 83% of the protein-encoding open reading frames in their respective genomes, but have slightly different carbohydrate metabolisms [50]. The best characterized of these is *T. maritima*, which has nearly seven percent of the predicted coding sequences in its genome (AE000512) dedicated to the catabolism and uptake of sugars and sugar polymers, including genes for two endoglucanases and several enzymes in the xylan degradation pathway [51]. Carbohydrate-active enzymes, including two cellulases, laminarase, xylanase, two possible β-D-xylosidases, α-D-glucuronidase, and α-L-arabinosidase have been purified from *T. maritima* [49]. Many of these genes have also been identified in the genomes of *T. petrophila* (CP000702) [52], *T. naphthophila* (CP001839), *T. neapolitana* (CP000916), and *T. thermarum* (CP002351). Other *Thermotoga* enzymes have been isolated and expressed recombinantly in *E. coli*, including pectate lyase [53], exopolygalacturonase [54], and α-L-arabinofuranosidase [55], though these genes may not all be expressed by *Thermotoga* [56], [57]. In addition to cellulose and hemicellulose depolymerization activity, *Thermotoga* species also express enzymes capable of hydrolyzing the disaccharide cellobiose [58] and the trisaccharide cellotriose [49]. They also ferment simple sugars, releasing H_2_, CO_2_, lactate, and acetate [59], suggesting that the *Thermotogae* were potentially involved in several steps of decomposition of the lignocellulosic substrates. The biology of *Thermotoga* implicates them in primary hemicellulolysis, but the inability of known strains to grow on crystalline cellulose suggests that other groups in the enrichments might be more important to the key step of primary cellulolysis.

### Enrichment of Heterotrophic Thermoprotei

Although the archaeal class *Thermoprotei* was abundant in both the enrichment samples (32.2 to 61.1% relative abundance) and the natural sediment (21.8% relative abundance) at Site 85, the *Thermoprotei* present in the enrichment samples were distinct from those found in 85 U. The *Thermoprotei* in the natural sediment sample were primarily affiliated with a single OTU, #C199, which shared 98% sequence identity over the pyrotag length with *Aeropyrum pernix* (NC_000854.2), a marine hyperthermophile that grows aerobically on a variety of proteinaceous compounds. This OTU accounted for 20.3% of the natural sediment community at Site 85, but no more than 4% of the pyrotags present in any enrichment sample. The *Thermoprotei* present in the enrichment samples at Site 85 included members of both the *Desulfurococcaceae* and *Thermofilaceae.* Two OTUs comprised most of the *Desulfurococcaceae*, #C011 and #C359. #C011 represented 3.9 to 9.8% of the microbial community in the Site 85 enrichment samples, where it showed an 821- to 2,080-fold enrichment over 85 U. OTU #C359 represented 15.6% to 23.5% of the community of the Site 85 enrichment samples, a 3,330- to 5,270-fold enrichment. #C359 shared 100% pyrotag sequence identity with an organism related to *Ignisphaera* that was identified within a lignocellulolytic consortium [60] enriched from GBS19, a ∼94°C geothermal spring, located ∼170 meters north of GBS within the Great Boiling Spring system and described in the supplementary material of [61]. The lignocellulolytic consortium was the first documented to degrade crystalline cellulose at temperatures exceeding 90°C. The *Ignisphaera*-like archaeon was identified as the microorganism responsible for the strong cellulolytic ability of the consortium and encoded a multi-domain cellulase with maximal activity at 109°C. The enrichment of these two OTUs on lignocellulosic material at Site 85 and the fact that the two OTUs shared high identity to an organism with demonstrated strong cellulolytic ability suggests that these microorganisms were likely participating directly in the primary decomposition of the lignocellulosic materials.

Members of *Thermofilaceae* at Site 85 included OTU #C867, which was 99% identical to *Thermofilum pendens* (CP000505) over the pyrotag length. OTU #C867 constituted less than 4.0% of the communities present in the aspen samples (85AS and 85AW), but represented 12.4% to 18.9% of the community of the corn stover samples (85CS and 85CW). *T. pendens* is a strictly anaerobic hyperthermophile that grows chemoorganotrophically using sulfur-based anaerobic respiration. The genome of *T. pendens* contains a large number of ABC transporters responsible for carbohydrate uptake, including a transporter with high similarity to a characterized cellobiose transporter, and genes dedicated to carbohydrate metabolism, including a secreted family of glycosyl hydrolases with weak similarity to known cellulases [62]. The organism represented by OTU #C867 may be responsible for some of the cellulolytic activity performed by the microbial communities in 85CS and 85CW, but based on the pronounced ability of *T. pendens* for carbohydrate uptake, it is more likely that this organism was enriched due to the release of saccharides by the cellulolytic and hemicellulolytic activities of other community members.

### Enrichment of Hemicellulolytic and Possibly Cellulolytic Dictyoglomi

Substrate-preferential enrichment was also observed for an OTU assigned to the phylum *Dictyoglomi*, the members of which are known to produce a variety of thermostable enzymes with significant biotechnological applications, including xylanases, amylases, and mannases [63]. The 16S rRNA genes of the only two described species of *Dictyoglomus*, *D. turgidum* (CP001251) and *D. thermophilum* (CP001146), share 99% sequence identity. The genus *Dictyoglomus* was represented in our samples almost entirely by a single OTU, #C692, which also shared 99% sequence identity to both *D. turgidum* and *D. thermophilum* over the length of the pyrotag. *D. turgidum* and *D. thermophilum* are strictly anaerobic and thermophilic chemoorganotrophs that can ferment a variety of carbohydrates. #C692 represented less than 0.4% of the 77U natural sediment sample, but was abundant in the enrichment samples at Site 77, except the anomalous 77CS. OTU #C692 showed greater enrichment in the Site 77 aspen samples, 77AW and 77AS, where it represented 18.3% to 20.6% of the community, than in the corn stover samples, where it represented only 9.7% of the 77CW community and 1.0% of the 77CS community. OTU #C692 was not detected in 85 U or the corn stover enrichments at Site 85, but did represent ∼2% of the aspen samples at Site 85, further illustrating this organism’s preference for the aspen material. Although the representative sequence of this OTU shared equal identity to both species of *Dictyoglomus*, its appearance in Site 85 samples is more consistent with the growth temperature range for *D. turgidum* (86°C maximum, 72°C optimum) [20], than with that of *D. thermophilum* (80°C maximum, 73–78°C optimum) [19]. *D. thermophilum* has not been shown to express cellulases, but does produce thermostable xylanases and can grow on a variety of fermentable carbohydrates, including several monohexoses and monopentoses, as well as cellobiose [19]. However, *D. turgidum* has been shown to utilize carboxymethylcellulose as a carbon source [20], [63], and the genes for 54 carbohydrate-active enzymes were annotated in its genome [64].

### Enrichment of Other Taxa

Also enriched were taxa with no or minimal known saccharolytic capability, including two OTUs within *Thermodesulfobacteria* (#C240 and #C707). Characterized *Thermodesulfobacteria* are known to use glycolysis intermediates and fermentation products such as pyruvate, lactate, and H_2_ as electron donors in the reduction of sulfate [65]. The organisms represented by these two OTUs were likely enriched by the metabolic products of other community members acting directly upon the enrichment substrates. OTU #C903, with ≥98% sequence identity to several members of the *Archaeoglobaceae* (AE000782, AJ299218, FJ216404, NR_028166, CP002588, CP001899, NR_041788, CP001857), was also enriched in all samples, showing an average 5.79-fold enrichment at Site 77 and representing an average 7.30% of the enriched community at Site 85. Characterized members of *Archaeoglobaceae* are known to use glucose as well as the fermentation products lactate and formate in the reduction of oxidized sulfur compounds [66], suggesting that this OTU may have been acting at multiple steps in the decomposition of lignocellulosic material. Also, because all known *Thermodesulfobacteria* and *Archaeoglobaceae* consume H_2_, these organisms might play a role in consortial lignocellulose fermentation by keeping concentrations of H_2_ low, reducing the inhibitory effects of H_2_ on fermentative processes.

Several members of candidate taxa were also enriched by the lignocellulosic substrates. OTU #C758, which belonged to the candidate bacterial phylum OP9, represented 7.1 to 9.5% of samples 77AS, 77AW, and 77CW, demonstrating a 51.4- to 69.4-fold increase over its representation in 77 U. OTU #C216, which was assigned to the candidate order pGrfC26 within the candidate archaeal class C2, was enriched in all aspen samples (56.8- to 235-fold enrichment) but was not detected in most corn stover samples. OTU #C896, identified as a member of archaeal candidate phylum DHVE3, showed a 12.8- to 16.3-fold enrichment in the aspen samples at Site 77 but did not appear in any other enrichments. Because these candidate taxa have not yet been described, their roles in the enriched community remain unclear, but their enrichment suggests that they are involved in consortial lignocellulose degradation and may represent unexplored sources of novel carbohydrate-active enzymes.

### Conclusions

Lignocellulose incubations in GBS stimulated the formation of distinct communities dominated by microorganisms found only in low abundance in the corresponding natural environment. The closest characterized relatives of the dominant enriched OTUs are organisms known to be capable of diverse metabolisms that together engage in consortial lignocellulose digestion, including cellulose and hemicellulose degraders, sugar fermenters, and hydrogenotrophs. Enriched communities were different at Site 85 and Site 77, primarily due to the lower taxonomic richness and higher representation of the archaeal classes *Thermoprotei* and *Archaeoglobi* at the higher-temperature site. *Thermotoga* were highly enriched in all samples at both sites, consistent with the utilization of a variety of carbohydrates by members of the genus. Two different species of *Thermotoga* were enriched in different samples and demonstrated apparent mutual exclusion. Several taxa showed preference for a specific lignocellulosic material; *Thermosphaera* and *Thermofilum* were more highly enriched on corn stover, whereas *Dictyoglomus*, *Thermodesulfobacteria*, and the archaeal group C2 showed marked preference for the aspen substrate.

Aspen and corn stover were consumed differently, with a higher ratio of hemicellulose degradation and possibly higher overall consumption of organic material in the corn stover samples than in the aspen samples. The specific reasons for this disparity are unclear, but the AFEX pretreatment and the higher initial ratio of hemicellulose to cellulose in the pretreated corn stover than in the aspen may have contributed to the differential degradation of each portion of the materials. Additional investigation, using a broader variety of lignocellulosic substrates, is necessary to fully elucidate the various factors leading to preferential decomposition of different components of lignocellulose by thermophilic communities.

The enzymatic conversion of lignocellulosic material to industrially useful materials such as ethanol and H_2_ involves several steps that are performed by several different organisms. This study characterized the thermophilic, cellulolytic and carbohydrate-active communities that form under several lignocellulose enrichment conditions in GBS and demonstrated the differential response of communities to different lignocellulose sources and incubation temperatures. Such factors are important for the optimization of industrial biofuel production processes. The differential decomposition of lignocellulosic stocks of varying cellulose and hemicellulose compositions and the temperature-specific formation of syntrophic communities comprised of mutually compatible organisms should inform the ongoing pursuit of systems for production of second-generation biofuels.

## Supporting Information

Figure S1 Log of iButton temperature loggers at sample incubation sites. Incubation of substrates began on 29 August, 2009, with temperatures at each incubation site indicated by green dots. Temperature-logging iButtons were deployed in the water column at incubation sites on 15 September, 2009. Temperatures were recorded every two hours from this date until the end of substrate incubation at each site (red diamonds). Logged temperature readings at each site are graphed with solid, colored lines. Black lines indicate the average temperatures of each site over the time logged.
(EPS)

Figure S2 Percent relative abundance of major bacterial phyla and archaeal classes in each sample. (A) Archaeal classes. (B) Bacterial phyla.
(EPS)

Figure S3 Alpha diversity measurements, comparing natural sediment and enrichment samples. (a) OTUs (≥97% identity) observed in each sample. (b) Simpson’s index of evenness in each sample.
(EPS)

Figure S4 Alpha diversity measurements, comparing natural and enrichment samples at 97%, 95%, 92%, 85%, and 80% OTU minimum identity levels. (a) OTUs observed in each sample. (b) Simpson’s index of evenness in each sample. Note U85 and U77 points are overlapping (same evenness scores) for 97% and 95% OTU identities.
(EPS)

Table S1 Quantification of DNA extracted from non-incubated and incubated substrates.
(DOC)

Table S2 Complete measurements of nutritional data for all samples.
(DOC)

Table S3 Counts of OTUs (≥97% identity) occurrence in each sample, including taxonomic identification of each OTU. Only OTUs with at least 1% relative abundance in at least one sample are included.
(DOC)

Table S4 PERMANOVA statistics indicating the significance of the difference between samples indicated by tree nodes and other logical differences between sample groups.
(DOC)

Table S5 SIMPER results for comparison of samples segregated at tree node #2, including U85, U77, and 77CS in one group and all other enrichment samples in the other group. Only OTUs contributing at least 1% of the difference of the community compositions are included.
(DOC)

Table S6 SIMPER results for comparison of samples from Site 85 to samples from Site 77, including natural sediment community samples at each site. Only OTUs contributing at least 1% of the difference of the community compositions are included.
(DOC)

Table S7 SIMPER results for comparison of natural sediment communities, U85 and U77, to all enrichment samples. Only OTUs contributing at least 1% of the difference of the community compositions are included.
(DOC)

Table S8 SIMPER results for comparison of samples segregated at tree node #3, including 77AS, 77AW, and 77CW in one group and all Site 85 enrichment samples in the other group. Only OTUs contributing at least 1% of the difference of the community compositions are included.
(DOC)
