# Supplementary material for: Pyrosequencing Reveals High-Temperature Cellulolytic Microbial Consortia in Great Boiling Spring after In Situ Lignocellulose Enrichment
Source: PLoS One. 2013 Mar 29;8(3):e59927. doi: 10.1371/journal.pone.0059927 (PMC3612082; doi:10.1371/journal.pone.0059927)
Supplement: Table S1 — Quantification of DNA extracted from non-incubated and incubated substrates. (DOC) [file pone.0059927.s005.doc]

| Table S1 | | |
| --- | --- | --- |
| Substrate and incubation | DNA | Fold increase over non-incubated ^a^ |
|  | (ng/g wet weight) |  |
| Non-incubated Aspen | 66 | na^b^ |
| 77AW | 840 | 12.7 |
| 77AS | 940 | 14.3 |
| 85AW | 1050 | 15.9 |
| 85AS | 1470 | 22.3 |
| Non-incubated Corn Stover | 1370 | na^b^ |
| 77CW | 3420 | 2.5 |
| 77CS | 4550 | 3.3 |
| 85CW | 3050 | 2.2 |
| 85CS | 7760 | 5.7 |
| ^a^ Compared within the given substrate type (aspen or corn stover) | | |
| ^b^ na, not applicable | | |
