# Supplementary material for: Pyrosequencing Reveals High-Temperature Cellulolytic Microbial Consortia in Great Boiling Spring after In Situ Lignocellulose Enrichment
Source: PLoS One. 2013 Mar 29;8(3):e59927. doi: 10.1371/journal.pone.0059927 (PMC3612082; doi:10.1371/journal.pone.0059927)
Supplement: Table S2 — Complete measurements of nutritional data for all samples. (DOC) [file pone.0059927.s006.doc]

| Table S2 | | | | | | | | | | |
| --- | --- | --- | --- | --- | --- | --- | --- | --- | --- | --- |
| **Label^1^** | **CP^2^** | **ADF^3^** | **NDF^4^** | **Lignin** | **ESC^5^** | **Ash^6^** | **Cell^7^** | **Hemicell^8^** | **NFC^9^** | **Other^10^** |
| 77AS | 1.9 | 77.8 | 91.8 | 13.3 | 0.1 | 9.6 | 64.5 | 14.0 | na^11^ | 0.0 |
| 77AW | 1.9 | 80.1 | 91.8 | 13.0 | 0.1 | 3.9 | 67.1 | 11.7 | na^11^ | 2.3 |
| 85AS | 1.9 | 75.5 | 85.7 | 14.0 | 1.3 | 11.5 | 61.5 | 10.2 | na^11^ | 0.0 |
| 85AW | 1.5 | 77.9 | 87.8 | 9.6 | 0.6 | 13.1 | 68.3 | 9.9 | na^11^ | 0.0 |
| UA | 1.7 | 73.5 | 89.3 | 12.9 | 0.9 | 0.8 | 60.6 | 15.8 | na^11^ | 7.4 |
| 77CS | 4.7 | 69.4 | 77.0 | 2.3 | 0.0 | 13.5 | 67.1 | 7.6 | 0.0 | na^11^ |
| 77CW | 4.6 | 80.0 | 88.7 | 8.2 | 0.7 | 9.8 | 71.8 | 8.7 | 5.0 | na^11^ |
| 85CS | 3.9 | 57.4 | 67.7 | 2.0 | 1.4 | 28.6 | 55.4 | 10.3 | 6.2 | na^11^ |
| 85CW | 4.8 | 67.9 | 76.4 | 4.9 | 3.0 | 15.1 | 63.0 | 8.5 | 0.0 | na^11^ |
| UC | 7.0 | 49.4 | 81.7 | 10.6 | 0.6 | 4.4 | 38.8 | 32.3 | 9.8 | na^11^ |
| ^1^ Label = temperature (77 or 85 °C), substrate (A= aspen shavings, C= corn stover), incubation location (S= sediment, W= water) | | | | | | | | | | |
| ^2^ Crude protein; in all samples, crude protein was equal to true protein | | | | | | | | | | |
| ^3^ Acid detergent fiber = cellulose + lignin | | | | | | | | | | |
| ^4^ Neutral Detergent Fiber = hemicellulose + cellulose + lignin | | | | | | | | | | |
| ^5^ Ethanol-soluble sugars (mostly mono- and di-saccharides) | | | | | | | | | | |
| ^6^ Mineral content or non-organic component | | | | | | | | | | |
| ^7^ Cellulose; calculated from ADF-Lignin | | | | | | | | | | |
| ^8^ Hemicellulose; calculated from NDF-ADF | | | | | | | | | | |
| ^9^ Non-fiber carbohydrates; %NFC = 100% - (%CP + (%NDF - %NDICP) + %Fat + %Ash). | | | | | | | | | | |
| ^10^ Other = components not analyzed, including fat, pectin, organic acids, and soluble fiber. | | | | | | | | | | |
| ^11^ na: Data not available. | | | | | | | | | | |
