# Supplementary material for: Pyrosequencing Reveals High-Temperature Cellulolytic Microbial Consortia in Great Boiling Spring after In Situ Lignocellulose Enrichment
Source: PLoS One. 2013 Mar 29;8(3):e59927. doi: 10.1371/journal.pone.0059927 (PMC3612082; doi:10.1371/journal.pone.0059927)
Supplement: Table S3 — Counts of OTUs (≥97% identity) occurrence in each sample, including taxonomic identification of each OTU. Only OTUs with at least 1% relative abundance in at least one sample are included. (DOC) [file pone.0059927.s007.doc]

| Table S3 | | | | | | | | | | | | |
| --- | --- | --- | --- | --- | --- | --- | --- | --- | --- | --- | --- | --- |
| **OTU** | **UW** | **U85** | **U77** | **77AS** | **77AW** | **77CS** | **77CW** | **85AS** | **85AW** | **85CS** | **85CW** | **Identity** |
| C004 | 0 | 177 | 229 | 1 | 1 | 26 | 4 | 0 | 0 | 0 | 0 | "*Aigarchaeota*" |
| C008 | 0 | 0 | 3 | 0 | 0 | 128 | 5 | 0 | 0 | 0 | 0 | *Thermus* sp. |
| C011 | 0 | 0 | 4 | 4 | 3 | 34 | 8 | 389 | 318 | 1256 | 1392 | *Ignisphaera*-like *Desulfurococcaceae* |
| C035 | 592 | 154 | 113 | 3 | 2 | 14 | 5 | 4 | 4 | 288 | 62 | *Pyrobaculum* *calidifontis* |
| C036 | 0 | 0 | 6 | 1 | 6 | 63 | 0 | 133 | 587 | 1317 | 373 | "*Aigarchaeota*" |
| C056 | 16 | 4053 | 513 | 0 | 0 | 31 | 19 | 0 | 0 | 244 | 35 | "*Aigarchaeota*" |
| C063 | 1 | 0 | 181 | 0 | 0 | 24 | 1 | 0 | 0 | 0 | 0 | GN03 (WS3) |
| C064 | 0 | 0 | 11 | 0 | 0 | 207 | 2 | 0 | 0 | 0 | 0 | *Rhodothermus* sp. |
| C121 | 0 | 0 | 199 | 210 | 37 | 0 | 0 | 26 | 26 | 0 | 0 | GAL35 |
| C136 | 5 | 2123 | 17 | 0 | 0 | 0 | 1 | 1 | 0 | 36 | 5 | Novel Archaeal Group I |
| C199 | 7 | 2940 | 418 | 0 | 0 | 33 | 2 | 0 | 5 | 408 | 32 | *Aeropyrum* sp. |
| C205 | 0 | 0 | 7 | 20 | 21 | 8 | 6 | 100 | 203 | 602 | 680 | *Desulfurococcus*-like *Desulfurococcaceae* |
| C206 | 0 | 0 | 0 | 33 | 42 | 76 | 51 | 33 | 49 | 28 | 84 | *Thermotoga* sp. |
| C216 | 0 | 0 | 6 | 900 | 621 | 0 | 5 | 53 | 23 | 0 | 0 | pGrfC26 (*Crenarchaeota* C2) |
| C235 | 0 | 1 | 70 | 58 | 20 | 0 | 0 | 3 | 2 | 0 | 0 | NRP-J (*Crenarchaeota*) |
| C236 | 10278 | 887 | 630 | 60 | 33 | 833 | 608 | 19 | 14 | 448 | 120 | *Thermocrinis* sp. |
| C240 | 1 | 18 | 55 | 385 | 287 | 22 | 80 | 396 | 279 | 105 | 128 | Unidentified Bacterium in *Thermodesulfobacteriaceae* |
| C242 | 0 | 0 | 0 | 56 | 105 | 8 | 17 | 5 | 9 | 0 | 0 | *Dictyoglomus* sp. |
| C245 | 1 | 85 | 329 | 0 | 0 | 9 | 4 | 0 | 1 | 1 | 0 | Unidentified Bacterium |
| C249 | 0 | 0 | 17 | 81 | 135 | 0 | 0 | 1 | 2 | 3 | 2 | *Geoglobus*-like *Archaeoglobaceae* |
| C253 | 0 | 0 | 16 | 0 | 1 | 2 | 2 | 13 | 6 | 152 | 62 | *Aeropyrum* sp. |
| C301 | 0 | 0 | 0 | 0 | 0 | 8 | 8 | 18 | 92 | 520 | 620 | *Thermosphaera* *aggregans* |
| C341 | 84 | 13 | 2 | 0 | 0 | 0 | 0 | 0 | 0 | 0 | 0 | Unidentified Bacterium in Aquificaceae |
| C359 | 0 | 0 | 0 | 0 | 3 | 70 | 0 | 758 | 2046 | 2906 | 5026 | *Ignisphaera*-like *Desulfurococcaceae* |
| C369 | 98 | 0 | 1 | 0 | 0 | 0 | 0 | 0 | 0 | 0 | 0 | *Tepidimonas* sp. |
| C422 | 0 | 1 | 127 | 1 | 1 | 1026 | 129 | 0 | 0 | 0 | 0 | Unidentified Bacterium in *Thermomicrobia* |
| C487 | 0 | 0 | 1437 | 1 | 3 | 390 | 42 | 0 | 1 | 0 | 0 | "*Aigarchaeota*" |
| C528 | 0 | 0 | 70 | 0 | 0 | 0 | 0 | 0 | 0 | 0 | 0 | LCP-6 (*Thermodesulfovibrionaceae*) |
| C529 | 0 | 0 | 12 | 678 | 147 | 2602 | 2100 | 1618 | 2900 | 1815 | 5633 | *Thermotoga* sp. |
| C543 | 0 | 0 | 152 | 0 | 0 | 0 | 0 | 0 | 0 | 0 | 0 | Unidentified Bacterium in GAL15 |
| C589 | 0 | 0 | 548 | 1 | 1 | 521 | 99 | 0 | 0 | 0 | 0 | Unidentified Bacterium in *Chlorobi* |
| C600 | 2 | 4 | 942 | 3 | 4 | 677 | 249 | 0 | 0 | 0 | 0 | *Candidatus* “Nitrosocaldus” sp. |
| C603 | 11 | 3727 | 2016 | 25 | 2 | 1765 | 995 | 13 | 33 | 270 | 103 | GAL35 |
| C628 | 0 | 0 | 10 | 376 | 231 | 0 | 0 | 5 | 75 | 0 | 1 | "*Aigarchaeota*" |
| C666 | 1 | 0 | 27 | 0 | 0 | 185 | 40 | 0 | 0 | 0 | 0 | *Thermus* *thermophilus* |
| C678 | 0 | 0 | 63 | 23 | 61 | 0 | 2 | 0 | 0 | 0 | 0 | *Fervidobacterium* sp. |
| C692 | 0 | 0 | 43 | 4327 | 3467 | 119 | 1022 | 91 | 184 | 0 | 0 | *Dictyoglomus* sp. |
| C707 | 0 | 12 | 46 | 491 | 573 | 3 | 25 | 280 | 83 | 8 | 9 | Unidentified Bacterium in *Thermodesulfobacteriales* |
| C716 | 0 | 0 | 72 | 118 | 2 | 0 | 0 | 0 | 0 | 0 | 0 | Unidentified Bacterium in *Syntrophobacteraceae* |
| C734 | 0 | 0 | 0 | 1 | 0 | 114 | 86 | 2 | 0 | 11 | 4 | GAL35 |
| C742 | 0 | 0 | 23 | 307 | 306 | 0 | 48 | 0 | 0 | 0 | 0 | *Caldicellulosiruptor* sp. |
| C743 | 0 | 0 | 3 | 0 | 1 | 0 | 0 | 77 | 34 | 0 | 8 | Unidentified Archaeon in *Thermoprotei* |
| C745 | 3 | 0 | 940 | 1 | 0 | 604 | 100 | 0 | 0 | 0 | 0 | OS-L (*Armatimonadetes*) |
| C758 | 0 | 0 | 15 | 1997 | 1801 | 179 | 745 | 4 | 0 | 1 | 0 | OPB72 (OP9) |
| C782 | 0 | 0 | 24 | 4613 | 6355 | 454 | 2394 | 15 | 7 | 0 | 0 | *Thermotoga* sp. |
| C790 | 0 | 0 | 12 | 2041 | 1370 | 19 | 342 | 0 | 0 | 0 | 0 | Unidentified Bacterium in *Gemmatimonadetes* |
| C800 | 0 | 0 | 111 | 2 | 2 | 0 | 0 | 2 | 1 | 0 | 0 | Unidentified Bacterium in *Thermodesulfobacteriaceae* |
| C840 | 0 | 0 | 93 | 32 | 17 | 1 | 5 | 30 | 67 | 0 | 0 | OP1 |
| C853 | 0 | 0 | 0 | 0 | 0 | 2 | 0 | 5 | 23 | 70 | 189 | *Thermofilum* *pendens* |
| C859 | 23 | 0 | 551 | 8 | 2 | 1226 | 137 | 0 | 0 | 1 | 0 | *Thermus* sp. |
| C867 | 0 | 0 | 5 | 0 | 0 | 21 | 0 | 142 | 309 | 1590 | 4027 | *Thermofilum* *pendens* |
| C887 | 0 | 1 | 0 | 4 | 2 | 2 | 0 | 124 | 43 | 6 | 16 | Unidentified Archaeon in DHVE3 |
| C890 | 0 | 0 | 6 | 546 | 355 | 2 | 43 | 25 | 46 | 0 | 0 | Unidentified Bacterium in EM3 |
| C896 | 0 | 0 | 6 | 154 | 177 | 0 | 0 | 0 | 0 | 0 | 0 | Unidentified Archaeon in DHVE3 |
| C898 | 0 | 0 | 216 | 0 | 0 | 86 | 8 | 0 | 0 | 0 | 0 | TK17 (*Chloroflexi*) |
| C903 | 0 | 0 | 157 | 2282 | 2025 | 222 | 937 | 306 | 594 | 556 | 2429 | *Archaeoglobus* sp. |
