# Supplementary material for: Pyrosequencing Reveals High-Temperature Cellulolytic Microbial Consortia in Great Boiling Spring after In Situ Lignocellulose Enrichment
Source: PLoS One. 2013 Mar 29;8(3):e59927. doi: 10.1371/journal.pone.0059927 (PMC3612082; doi:10.1371/journal.pone.0059927)
Supplement: Table S4 — PERMANOVA statistics indicating the significance of the difference between samples indicated by tree nodes and other logical differences between sample groups. (DOC) [file pone.0059927.s008.doc]

| **Table S4** | | |
| --- | --- | --- |
| **Variation** | **F^a^** | **p-value** |
| Node 2: Natural sediment and 77CS vs. Enrichment samples | 4.375 | 0.0084 ** |
| Site: 85 vs. 77, incl. natural sediment samples. | 3.475 | 0.0152 * |
| Enriched :Enrichment vs. natural samples | 3.606 | 0.0199 * |
| Node 3: 77 (exc. 77CS) vs. 85, enrichment samples only | 15.63 | 0.0275 * |
| Node 1: UW vs. all others | 2.077 | 0.0892 ~ |
| Enrichment Corn stover enrichments vs. aspen enrichments) | 1.454 | 0.1765 |
| Node 7: Site 85 aspen vs. Site 85 corn stover | 3.525 | 0.3245 |
| Node 6: Site 77 aspen vs. 77CW | 9.623 | 0.3354 |
| Node 4: U85 vs. U77 and 77CS | 2.321 | 0.3413 |
| Location: Sediment enrichments vs. water enrichments | 0.2102 | 0.8306 |
| ** Significant at α = 0.01 |  |  |
| * Significant at α = 0.05 |  |  |
| ~ Significant at α = 0.1 |  |  |
| ^a^ PERMANOVA F-Statistic |  |  |
