# Supplementary material for: Pyrosequencing Reveals High-Temperature Cellulolytic Microbial Consortia in Great Boiling Spring after In Situ Lignocellulose Enrichment
Source: PLoS One. 2013 Mar 29;8(3):e59927. doi: 10.1371/journal.pone.0059927 (PMC3612082; doi:10.1371/journal.pone.0059927)
Supplement: Table S5 — SIMPER results for comparison of samples segregated at tree node #2, including U85, U77, and 77CS in one group and all other enrichment samples in the other group. Only OTUs contributing at least 1% of the difference of the community compositions are included. (DOC) [file pone.0059927.s009.doc]

| **Table S5** | | | | | |
| --- | --- | --- | --- | --- | --- |
| **OTU** | **Identity** | **Δ^a^** | **Contrib. (%)^b^** | **Avg. Rep. (%) U85, U77, 77CS ^c^** | **Avg. Rep. (%) Enr. Exc. 77CS ^d^** |
| C603 | GAL35 | - | 10.31 | 19.64 | 1.82 |
| C529 | *Thermotoga* sp. | + | 9.428 | 7.08 | 18.98 |
| C359 | *Ignisphaera*-like *Desulfurococcaceae* | + | 7.087 | 0.25 | 12.29 |
| C782 | *Thermotoga* sp. | + | 6.629 | 1.38 | 11.34 |
| C056 | *"Aigarchaeota"* | - | 6.381 | 11.17 | 0.29 |
| C199 | *Aeropyrum* sp. | - | 4.407 | 7.83 | 0.55 |
| C903 | *Archaeoglobus* sp. | + | 4.367 | 1.01 | 8.55 |
| C692 | *Dictyoglomus* sp. | + | 4.176 | 0.43 | 7.39 |
| C867 | *Thermofilum pendens* | + | 3.165 | 0.10 | 5.49 |
| C487 | *"Aigarchaeota"* | - | 3.07 | 5.33 | 0.05 |
| C859 | *Thermus* sp. | - | 2.953 | 5.16 | 0.18 |
| C136 | Novel Archaeal Group I | - | 2.743 | 4.73 | 0.06 |
| C236 | *Thermocrinis* sp. | - | 2.668 | 6.15 | 1.61 |
| C600 | *Candidatus* “Nitrosocaldus” sp. | - | 2.592 | 4.61 | 0.36 |
| C745 | OS-L (*Armatimonadetes*) | - | 2.557 | 4.46 | 0.13 |
| C011 | *Ignisphaera*-like *Desulfurococcaceae* | + | 2.302 | 0.09 | 3.99 |
| C758 | OPB72 (OP9) | + | 2.093 | 0.52 | 3.57 |
| C422 | Unidentified Bacterium in *Thermomicrobia* | - | 1.857 | 3.24 | 0.18 |
| C036 | *"Aigarchaeota"* | + | 1.803 | 0.19 | 3.16 |
| C589 | Unidentified Bacterium in *Chlorobi* | - | 1.713 | 3.02 | 0.14 |
| C790 | Unidentified Bacterium in *Gemmatimonadetes* | + | 1.708 | 0.08 | 2.93 |
| C240 | Unidentified Bacterium in *Thermodesulfobacteriaceae* | + | 1.309 | 0.23 | 2.50 |
| C707 | Unidentified Bacterium in *Thermodesulfobacteriales* | + | 1.021 | 0.17 | 1.86 |
| ^a^ Difference between natural sediment and enrichment populations. + OTU has greater representation in enrichment samples (exc. 77CS). - OTU has lower representation in enrichment samples (exc. 77CS). | | | | | |
| ^b^ Percent contribution to community composition difference | | | | | |
| ^c^ Average percent representation in natural sediment sample communities and 77CS | | | | | |
| ^d^ Average percent representation in enrichment sample communities exc. 77CS | | | | | |
