# Supplementary material for: Pyrosequencing Reveals High-Temperature Cellulolytic Microbial Consortia in Great Boiling Spring after In Situ Lignocellulose Enrichment
Source: PLoS One. 2013 Mar 29;8(3):e59927. doi: 10.1371/journal.pone.0059927 (PMC3612082; doi:10.1371/journal.pone.0059927)
Supplement: Table S6 — SIMPER results for comparison of samples from Site 85 to samples from Site 77, including natural sediment community samples at each site. Only OTUs contributing at least 1% of the difference of the community compositions are included. (DOC) [file pone.0059927.s010.doc]

| **Table S6** | | | | | |
| --- | --- | --- | --- | --- | --- |
| **OTU** | **Identity** | **Δ^a^** | **Contrib. (%)^b^** | **Avg. Rep. (%) Site 85 ^c^** | **Avg. Rep. (%) Site 77 ^d^** |
| C529 | *Thermotoga* sp. | - | 10.56 | 21.69 | 9.05 |
| C359 | *Ignisphaera*-like *Desulfurococcaceae* | - | 10.44 | 17.20 | 0.15 |
| C782 | *Thermotoga* sp. | + | 10.08 | 0.09 | 16.60 |
| C603 | GAL35 | + | 6.205 | 5.89 | 8.47 |
| C692 | *Dictyoglomus* sp. | + | 5.768 | 0.80 | 9.83 |
| C867 | *Thermofilum pendens* | - | 4.662 | 7.68 | 0.06 |
| C056 | “*Aigarchaeota*” | - | 3.958 | 6.07 | 1.03 |
| C011 | *Ignisphaera*-like *Desulfurococcaceae* | - | 3.385 | 5.58 | 0.06 |
| C758 | OPB72 (OP9) | + | 3.208 | 0.02 | 5.29 |
| C903 | *Archaeoglobus* sp. | + | 2.97 | 5.89 | 6.69 |
| C199 | *Aeropyrum* sp. | - | 2.947 | 4.71 | 0.75 |
| C036 | “*Aigarchaeota*” | - | 2.641 | 4.40 | 0.12 |
| C790 | Unidentified Bacterium in *Gemmatimonadetes* | + | 2.54 | 0.00 | 4.17 |
| C859 | *Thermus* sp. | + | 2.044 | 0.00 | 3.35 |
| C236 | *Thermocrinis* sp. | + | 1.989 | 2.09 | 3.86 |
| C487 | “*Aigarchaeota*” | + | 1.988 | 0.00 | 3.26 |
| C600 | *Candidatus* “Nitrosocaldus” sp. | + | 1.986 | 0.01 | 3.26 |
| C136 | Novel Archaeal Group I | - | 1.771 | 2.91 | 0.03 |
| C745 | OS-L (*Armatimonadetes*) | + | 1.741 | 0.00 | 2.85 |
| C240 | Unidentified Bacterium in *Thermodesulfobacteriaceae* | - | 1.46 | 2.64 | 0.99 |
| C205 | *Desulfurococcus*-like *Desulfurococcaceae* | - | 1.422 | 2.38 | 0.09 |
| C422 | Unidentified Bacterium in *Thermomicrobia* | + | 1.338 | 0.00 | 2.19 |
| C589 | Unidentified Bacterium in *Chlorobi* | + | 1.222 | 0.00 | 2.00 |
| C707 | Unidentified Bacterium in *Thermodesulfobacteriales* | - | 1.143 | 1.44 | 1.26 |
| C301 | *Thermosphaera aggregans* | - | 1.019 | 1.69 | 0.02 |
| ^a^ Difference between populations at different sites. + OTU has greater representation in Site 77 samples. - OTU has greater representation in Site 85 samples. | | | | | |
| ^b^ Percent contribution to community composition difference | | | | | |
| ^c^ Average percent representation in Site 85 samples | | | | | |
| ^d^ Average percent representation in Site 77 samples | | | | | |
