# Supplementary material for: Pyrosequencing Reveals High-Temperature Cellulolytic Microbial Consortia in Great Boiling Spring after In Situ Lignocellulose Enrichment
Source: PLoS One. 2013 Mar 29;8(3):e59927. doi: 10.1371/journal.pone.0059927 (PMC3612082; doi:10.1371/journal.pone.0059927)
Supplement: Table S7 — SIMPER results for comparison of natural sediment communities, U85 and U77, to all enrichment samples. Only OTUs contributing at least 1% of the difference of the community compositions are included. (DOC) [file pone.0059927.s011.doc]

| Table S7 | | | | | |
| --- | --- | --- | --- | --- | --- |
| **OTU** | **Identity** | **Δ^a^** | **Contrib. (%)^b^** | **Avg. Env. Rep. (%)^c^** | **Avg. Enr. Rep. (%)^d^** |
| C529 | *Thermotoga* sp. | + | 10.76 | 0.04 | 19.25 |
| C603 | GAL35 | - | 10.36 | 21.89 | 3.47 |
| C056 | *"Aigarchaeota"* | - | 9.18 | 16.67 | 0.28 |
| C199 | *Aeropyrum* sp. | - | 6.22 | 11.61 | 0.51 |
| C359 | *Ignisphaera*-like *Desulfurococcaceae* | + | 6.08 | 0.00 | 10.84 |
| C782 | *Thermotoga* sp. | + | 5.81 | 0.10 | 10.41 |
| C136 | Novel Archaeal Group I | - | 3.97 | 7.10 | 0.06 |
| C903 | *Archaeoglobus* sp. | + | 3.95 | 0.65 | 7.70 |
| C692 | *Dictyoglomus* sp. | + | 3.66 | 0.13 | 6.61 |
| C487 | *"Aigarchaeota"* | - | 3.62 | 6.46 | 0.42 |
| C867 | *Thermofilum pendens* | + | 2.71 | 0.02 | 4.83 |
| C745 | OS-L (*Armatimonadetes*) | - | 2.45 | 4.38 | 0.69 |
| C600 | *Candidatus* “Nitrosocaldus” sp. | - | 2.37 | 4.25 | 0.98 |
| C236 | *Thermocrinis* sp. | - | 2.17 | 5.78 | 2.27 |
| C011 | *Ignisphaera*-like *Desulfurococcaceae* | + | 1.98 | 0.00 | 3.53 |
| C758 | OPB72 (OP9) | + | 1.84 | 0.09 | 3.28 |
| C859 | *Thermus* sp. | - | 1.77 | 2.44 | 1.49 |
| C036 | *"Aigarchaeota"* | + | 1.58 | 0.02 | 2.83 |
| C790 | Unidentified Bacterium in *Gemmatimonadetes* | + | 1.45 | 0.06 | 2.58 |
| C589 | Unidentified Bacterium in *Chlorobi* | - | 1.34 | 2.40 | 0.65 |
| C240 | Unidentified Bacterium in *Thermodesulfobacteriaceae* | + | 1.10 | 0.28 | 2.21 |
| C245 | Unidentified Bacterium | - | 1.03 | 1.85 | 0.02 |
| ^a^ Difference between natural sediment and enrichment populations. + OTU has greater representation in enrichment samples. - OTU has lower representation in enrichment samples. | | | | | |
| ^b^ Percent contribution to community composition difference | | | | | |
| ^c^ Average percent representation in natural sediment sample communities | | | | | |
| ^d^ Average percent representation in enrichment sample communities | | | | | |
